# Cardiovascular Abnormalities and Gene Mutations in Children With Noonan Syndrome

**DOI:** 10.3389/fgene.2022.915129

**Published:** 2022-06-13

**Authors:** Ling Sun, Yu-mei Xie, Shu-shui Wang, Zhi-wei Zhang

**Affiliations:** Department of Pediatric Cardiology, Guangdong Cardiovascular Institute, Guangdong Provincial People’s Hospital, Guangdong Academy of Medical Sciences, Guangzhou, China

**Keywords:** cardiovascular abnormalities, echocardiography, genotype-phenotype, noonan syndrome, pulmonary valve stenosis

## Abstract

**Background:** Common cardiac abnormalities in Noonan syndrome (NS) include congenital heart diseases (CHD), pulmonary valve stenosis and hypertrophic cardiomyopathy (HCM). Molecular diagnoses are enabling earlier and more precise diagnosis of patients who have a subtle or atypical presentation. The aims of this study were to investigate genotype-phenotype associations with respect to Noonan syndrome (NS)-associated cardiac abnormalities and catheter or surgery-based interventions conditions.

**Methods:** From January 2019 to December 2021, 22 children with a confirmed molecular diagnosis of NS combined with cardiovascular abnormalities were consecutively enrolled into the current study. A comprehensive review was carried out of echocardiography and electrocardiogram results, second-generation whole-exome sequencing results and catheter or surgery-based interventions conditions.

**Results:** The main manifestations of electrocardiogram abnormalities were QTc prolongation, abnormal Q wave in the precordial lead and limb lead, right ventricular hypertrophy and left or right deviation of the electrical axis. The most commonly detected abnormality was pulmonary valve dysplasia with stenosis, seen in 15 (68.2%) patients, followed by atrial septal defect in 11 (50%) patients. Seven genes (*RAF1*, *RIT1*, *SOS1*, *PTPN11*, *BRAF*, *SOS2*, and *LZTR1*) were found to contain disease-associated variants*.* The most commonly observed genetic mutations were *PTPN11* (27%) and *RAF1* (27%)*.* Each genotype was associated with specific phenotypic findings. *RIT1*, *SOS1*, *PTPN11*, and *SOS2* had common echocardiography features characterized by pulmonary valve stenosis, while *RAF1* was characterized by HCM. Interestingly, patients with *BRAF* mutations were not only characterized by HCM, but also by pulmonary valve stenosis. In the cohort there was only one patient carrying a *LZTR1* mutation characterized by left ventricle globose dilation. Ten cases underwent catheter or surgery-based interventions. All the operations had immediate results and high success rates. However, some of the cases had adverse outcomes during extended follow-up. Based on the genotype-phenotype associations observed during follow-up, *BRAF* and *RAF1* genotypes seem to be poor prognostic factors, and multiple interventions may be required for NS patients with severe pulmonary stenosis or myectomy for HCM.

**Conclusions:** The identification of causal genes in NS patients has enabled the evaluation of genotype-cardiac phenotype relationships and prognosis of the disease. This may be beneficial for the development of therapeutic approaches.

## Introduction

Noonan syndrome (NS) is an autosomal dominant disorder that is characterized by facial dysmorphism (hypertelorism, ptosis and low-set ears), short stature, congenital heart defects and other additional extracardiac features (Romano et al., 2010). Although Noonan syndrome is mainly transmitted as a dominant trait, an autosomal recessive form of this condition has recently been associated with biallelic LZTR1 variants (Johnston et al., 2018). The incidence of NS is estimated to be 1 in 1000–2500 live births (Allanson et al., 1985). One of the principal features of NS, which is also the cause of seeking early treatment, is cardiac involvement, most frequently including congenital heart diseases, pulmonary valve stenosis and hypertrophic cardiomyopathy (HCM) (Romano et al., 2010). With the elucidation of the underlying genetic variants causing NS, numerous studies have revealed that NS and NS-like disorders are ‘RASopathies’ or ‘RAS/mitogen-activated protein kinase (MAPK) syndromes’ because they share aberrant signaling through the RAS/MAPK pathway that controls cell proliferation, differentiation and survival (Aoki et al., 2008; Tidyman and Rauen, 2009). Recently, some studies have discussed the genotype-phenotype associations together with the prognostic assessment in these patients. A large study was published by Calcagni et al. in 2020. The authors reported the cardiac features in 440 molecularly characterized patients with RASopathies enrolled from seven centers participating in the CArdiac Rasopathy NETwork—CARNET study (Calcagni et al., 2020). Among them, 312 subjects presented a common congenital heart defect or HCM, whereas atypical findings were reported in 45 patients. Leoni et al. assessed genotype-cardiac phenotype correlations in a single-center cohort of 116 patients with a molecularly confirmed RASopathy (Leoni et al., 2022). Yi et al. assessed the therapeutic landscape for the treatment of heart disease in RASopathies (Yi et al., 2022). In addition, the clinical presentation and natural history of RASopathy-related HCM was extensively described in two recent reviews (Calcagni et al., 2018; Lioncino et al., 2022). To evaluate genotype-cardiac phenotype relationships and improve prognoses for patients with NS, it is necessary to look for genotype-phenotype associations with respect to NS-associated congenital heart diseases (CHD), and a personalized targeted therapy approach should be developed according to genotype-phenotype correlations.

In this study, we reviewed the cardiac manifestations of patients with a confirmed molecular diagnosis of NS by evaluating clinical echocardiography and electrocardiogram features and performing molecular analyses of the known causative genes of NS. We also evaluated genotype-cardiac phenotype associations.

## Methods

### Study Participants

This retrospective study was approved by the Clinical Research Ethic Committee of Guangdong Provincial People’s Hospital (ethical approval number KY-Z-2021-191-01 and KY2020-033-01). From January 2019 to December 2021, 22 children with a confirmed molecular diagnosis of NS combined with cardiovascular abnormalities were consecutively enrolled into the current study after parental written informed consent was obtained. Some of the children underwent corrective cardiac surgery or catheter-based interventions at Guangdong Provincial People’s Hospital, the largest tertiary cardiac center in China. We reviewed the clinical phenotypes of cardiac structural anomalies evaluated by electrocardiogram, echocardiography findings and follow-up data.

### Data Collection

Transthoracic echocardiographic examination was carried out preoperatively using IE33, IE Epic-7C (Philips, Andover, MA, United States) and Vivid E 9 (GE Healthcare, Horten, Norway) ultrasound systems with a transducer of 5–8 Mhz. The preoperative and postoperative TTE images and electrocardiogram were reviewed by two independent experienced pediatric cardiologists. If there were discrepancies between these two observers, another independent experienced pediatric cardiologist was invited to review the images.

### Molecular Genetic Analyses

Molecular diagnosis was performed using second-generation whole-exome sequencing. Genomic DNA was extracted from peripheral blood using the Solpure Blood DNA kit (Magen), then fragmented by Q800R Sonicator (Qsonica) to generate 200–500 bp insert fragments. Custom designed NimbleGen SeqCap probes (Roche NimbleGen, Madison, Wis) were used for in-solution hybridization to enrich target sequences. Enriched DNA samples were indexed and sequenced on a NextSeq500 sequencer (Illumina, San Diego, Calif) with 100 cycles of single end reads, according to the manufacturer’s protocols. The target gene capture/NGS assay provides an average read depth of approximately 200×. The insufficiently covered (<20×) target exons were analyzed by using gene-specific amplicon-based Sanger sequencing. Deleterious mutations and novel variants detected by using NGS were confirmed by means of Sanger sequencing. Variants interpretation was manipulated according to the American College of Medical Genetics (ACMG) guidelines (Richards et al., 2015). When an NS molecular diagnosis report was suspected, a clinical geneticist was invited to evaluate the diagnosis. The NS patients and their molecular diagnoses are shown in Table 1.

**TABLE 1:** NS patients and their clinical characteristics.

Case	Sex (F/M)	Age (m)	Genotype	Nucleotide change	Origin of variant	ACMG	Echo prestation	ECG	Follow-up (y)
Case 1	F	76	*RAF1*	c.770C > T	New mutations	1	Obstructive HCM, PFO, ASD, the right coronary artery arises from the left coronary sinus with intramural course	QTc prolonged, V4-V6 leads abnormal Q wave, electrical axis deviation to the right	2
Case 2	F	10	*RIT1*	c.229G > A	New mutations	1	Pulmonary valve dysplasia with stenosis (moderate to severe); biventricular hypertrophy; PFO	Right ventricular hypertrophy	2.5
Case 3	F	10	*SOS1*	c.508A > G	New mutations	1	Pulmonary valve dysplasia with stenosis (severe); biventricular hypertrophy; ASD; left pulmonary artery slight stenosis	Right ventricular hypertrophy	2
Case 4	M	15	*RIT1*	c.170C > G	New mutations	1	Pulmonary valve dysplasia with stenosis (severe); biventricular hypertrophy; ASD; PFO; slightly dilated opening of the left coronary artery	Atrial hypertrophy, left and right ventricular hypertrophy	1
Case 5	F	16	*SOS1*	c.770C > T	New mutations	1	Pulmonary valve dysplasia with stenosis (moderate); biventricular hypertrophy	Right ventricular hypertrophy	1.5
Case 6	F	11	*PTPN11*	c.174C > G	New mutations	2	Pulmonary valve stenosis (mild); PFO; left pulmonary artery stenosis	Normal	No
Case 7	M	23	*PTPN11*	c.922A > G	New mutations	1	Pulmonary valve stenosis (mild); ASD	Normal	No
Case 8	F	36	*RAF1*	c.770C > T	New mutations	1	Obstructive HCM; mitral regurgitation (moderate-severe)	QTc prolonged, electric axis deviated to the left, T wave changed	1.25
Case 9	M	96	*PTPN11*	c.922A > G	New mutations	1	Pulmonary valve bicuspid with regurgitation; ASD; tricuspid regurgitation; right aortic coronary valve prolapse with regurgitation	Sinus arrhythmia, electric axis deviated to the left, I, avL leads abnormal Q wave	0.5
Case 10	M	20	*PTPN11*	c.188A > G	New mutations	1	Pulmonary valve stenosis (mild); ASD	Normal	1
Case 11	M	0.7	*RAF1*	c.770C > T	New mutations	1	Left ventricular hypertrophy; ASD	Frequent atrial premature beats	No
Case 12	F	7	*RIT1*	c.284G > C	New mutations	1	Pulmonary valve stenosis with regurgitation (mild); ASD; PFO	Right ventricular hypertrophy	3
Case 13	M	23	*BRAF*	c.1502A > G	New mutations	2	Pulmonary valve stenosis (severe); mitral regurgitation (mild-moderate); biventricular hypertrophy; ASD	Atrial hypertrophy, left and right ventricular hypertrophy	1.5
Case 14	F	49	*SOS2*	c.458G > A	New mutations	2	Pulmonary valve stenosis (severe) with regurgitation (mild)	Right ventricular hypertrophy	0.25
Case 15	F	5	*BRAF*	c.1796C > G	New mutations	1	Pulmonary valve stenosis (mild)	Normal	No
Case 16	M	22	*LZTR1*	c.742G > A	New mutations	1	Left ventricle globose dilated	Normal	2
Case 17	F	108	*BRAF*	c.736G > C	New mutations	2	Obstructive HCM; mitral regurgitation (severe); tricuspid regurgitation (mild)	Normal	No
Case 18	M	12	*PTPN11*	c.922A > G	New mutations	1	No obstructive HCM; ASD; PFO	Normal	1
Case 19	M	66	*RAF1*	c.775T > A	New mutations	1	Obstructive HCM; mitral regurgitation (mild)	Normal	1
Case 20	F	168	*RAF1*	c.1082G > C	New mutations	2	Obstructive HCM; mitral regurgitation (mild)	Sinus bradycardia with irregularities,Ⅱ,Ⅲ, aVF abnormal Q wave	No
Case 21	M	36	*PTPN11*	c.124A > G	New mutations	1	Pulmonary valve stenosis (severe); VSD, ASD, PDA	Complete right bundle branch block, right ventricular hypertrophy	2
Case 22	M	60	*RAF1*	c.775T > A	New mutations	1	Left ventricular hypertrophy; mitral regurgitation (severe); tricuspid regurgitation (mild)	No	No

Abbreviations: HCM, hypertrophic cardiomyopathy; PFO, patent foramen ovale; ASD, atrial septal defect; PDA, patent ductus arteriosus; VSD, ventricular septal defect; ACMG, american college of medical genetics.

### Statistical Analysis

Continuous variables are presented as mean ± standard deviation if normally distributed, otherwise they are presented as the median (interquartile range). Categorial variables are presented as number and proportion. All analyses were performed using SPSS Statistics 23 software (IBM, Chicago, IL, United States).

## Results

### Electrocardiographic Findings

A total of 22 children with NS were included in the current analysis. There were 11 female patients and 10 male patients. Their ages ranged from 0.7 to 168 months old (mean age: 39.5 ± 41.6 months). All patients had confirmed diagnoses by molecular analysis. The preoperative echocardiography and electrocardiogram features and genotypes are listed in Table 1. Follow-up ranged from 0.5 to 3 years (mean follow-up: 1.02 ± 0.94 years). Case 22 had no follow-up and no electrocardiogram. Thirteen cases (62%) had electrocardiographic abnormalities. The main manifestations of electrocardiogram abnormalities were QTc prolongation, abnormal Q wave in the precordial lead and limb lead, right ventricular hypertrophy and left or right deviation of the electrical axis.

### Cardiac Abnormalities

**FIGURE 1:**
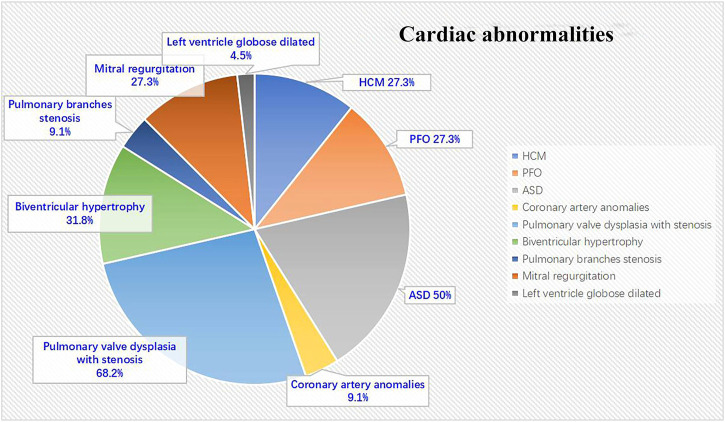
Cardiac abnormalities in NS patients (N = 22).

All 22 NS patients had cardiac abnormalities. The most commonly detected abnormality was pulmonary valve dysplasia with stenosis, seen in 15 (68.2%) patients, followed by atrial septal defect (ASD) in 11 (50%) patients, biventricular hypertrophy in seven (31.8%) patients, left HCM in six (27.3%) patients, mitral regurgitation in six (27.3%) patients, patent foramen ovale (PFO) in six (27.3%) patients, both coronary artery anomalies and pulmonary branches stenosis in two (9.1%) patients and left ventricle globose dilated in one (4.5%) patient (Figure 1).

### Genotype–Cardiac Phenotype Abnormalities

**FIGURE 2:**
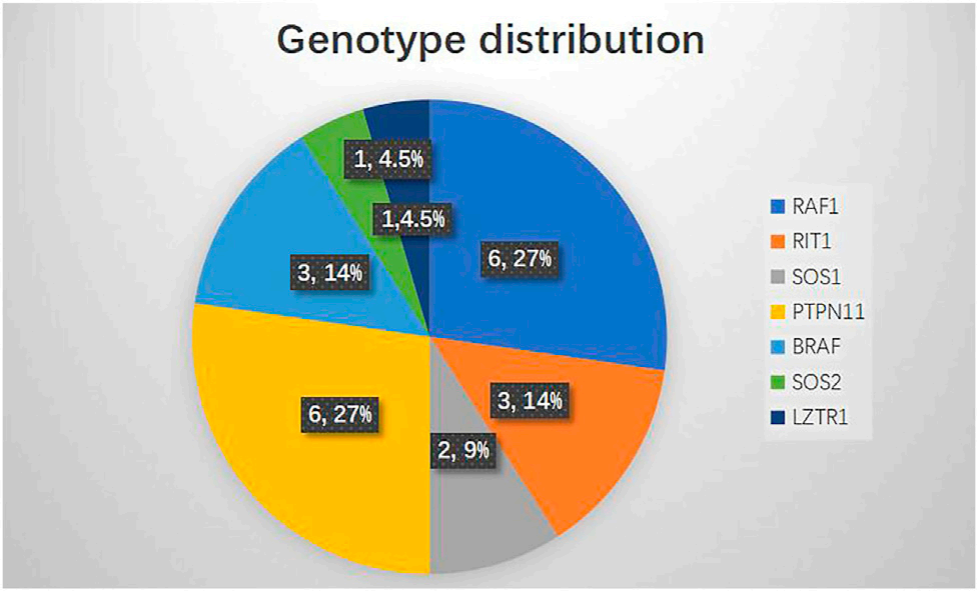
Genotype distribution in NS patients (N = 22).

The genotype distribution in the 22 NS patients is listed in Figure 2, including mutation of *RAF1* in six patients (27%), *RIT1* in three patients (14%), *SOS1* in two patients (9%), *PTPN11* in six patients (27%), *BRAF* in three patients (14%), *SOS2* in one patient (4.5%) and *LZTR1* in one patient (4.5%). The most commonly observed genetic mutations in patients were in *PTPN11* and *RAF1.* Each genotype had specific echocardiography findings. Patients with *RAF1* mutations were characterized by left ventricular hypertrophy (obstructive or not obstructive), ASD and mitral regurgitation. Patients with *RIT1* mutation were characterized by pulmonary valve dysplasia with stenosis and ASD/PFO. Patients with mutations in *SOS1* had pulmonary valve dysplasia with stenosis, biventricular hypertrophy and ASD. *PTPN11*-mutation carriers had pulmonary valve stenosis and ASD. *BRAF* carriers had pulmonary valve stenosis, obstructive HCM, ASD, and mitral regurgitation. *SOS2*-mutation carriers had pulmonary valve stenosis with regurgitation. Only one patient carrying a LZTR1 mutation had left ventricle globose dilation (Table 2). Among all the pathogenic variants, we found that patients with mutations in *RIT1*, *SOS1*, *PTPN11*, and *SOS2* had common echocardiography figures characterized by pulmonary valve stenosis, while *RAF1* patients had HCM. Interestingly, *BRAF* patients were characterized not only by HCM but also by pulmonary valve stenosis, meaning poor prognoses.

**TABLE 2:** Genotype–cardiac phenotype abnormalities in NS patients (N = 22).

Genotype	Cardiac Echo prestation	N (%)
*RAF1*	Left ventricular hypertrophy (obstructive or un- obstructive), ASD, mitral regurgitation	6 (27)
*RIT1*	Pulmonary valve dysplasia with stenosis, ASD/PFO	3 (14)
*SOS1*	Pulmonary valve dysplasia with stenosis, biventricular hypertrophy, ASD	2 (9)
*PTPN11*	Pulmonary valve stenosis, ASD	6 (27)
*BRAF*	Pulmonary valve stenosis, Obstructive HCM, ASD, mitral regurgitation	3 (14)
*SOS2*	Pulmonary valve stenosis with regurgitation	1 (4.5)
*LZTR1*	Left ventricle globose dilated	1 (4.5)

Abbreviations: HCM, hypertrophic cardiomyopathy; PFO, patent foramen ovale; ASD, atrial septal defect.

### Catheter or Surgery-Based Interventions and Follow-Up Data

**FIGURE 3:**
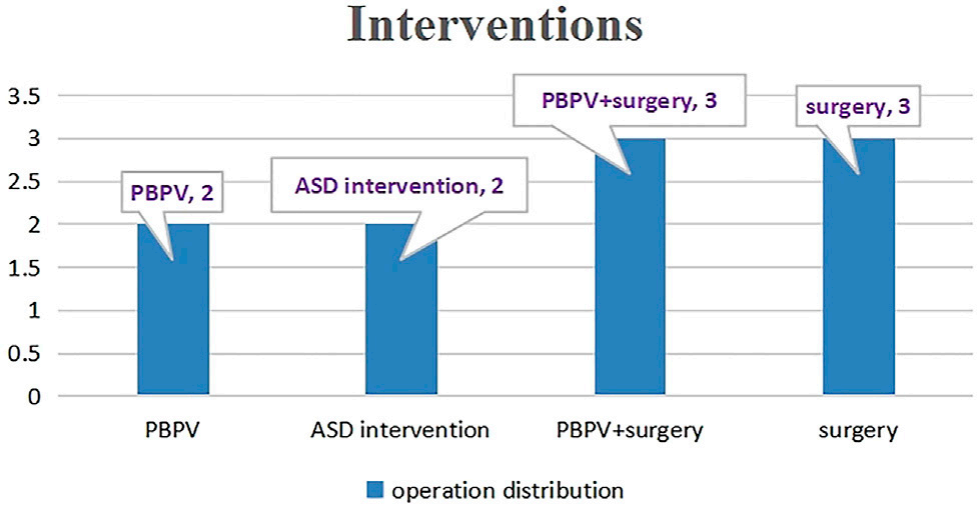
Operation distributions in interventions patients.

**FIGURE 4:**
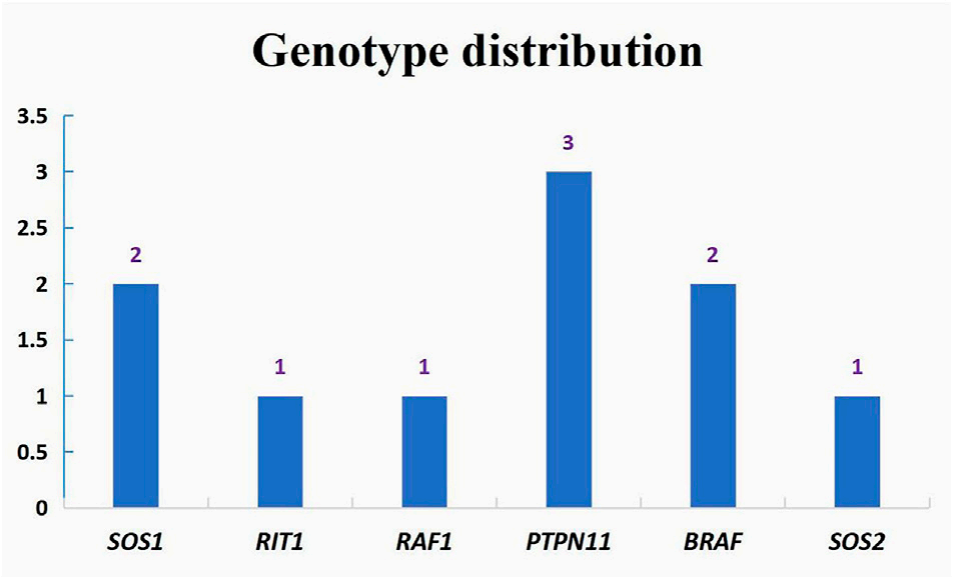
Genotype distribution in interventions patients.

A total of ten cases underwent catheter or surgery-based interventions (Table 3). Percutaneous balloon pulmonary valve dilation (PBPV) was seen in two (20%) patients, percutaneous closure of ASD in two (20%) patients and complex surgery in three (30%) patients. Another three cases underwent not only PBPV, but also complex surgery because of poor outcomes (Figure 3). The genotype distribution in 10 NS patients is listed in Figure 4, including *SOS1* in two patients (20%), *RIT1* in one patient (10%), *RAF1* in one patient (10%), *PTPN11* in three patients (30%), *BRAF* in two patients (20%) and *SOS2* in one patient (10%). All surgeries had immediate results and high success rates. However, some of the cases had adverse outcomes during extended follow-up. According to the follow-up results of the last echocardiography, the degree of pulmonary valve stenosis and the left ventricular outflow tract severity were determined from measurements of mean and peak gradients across the obstruction, and were quantitatively divided into mild, medium, and severe grades, following the recommendations of ESC guidelines (Baumgartner et al., 20202021) and EAE/ASE (Baumgartner et al., 2009). *SOS1, RIT1, PTPN11*, and *SOS2* mutations seemed to have good prognosis, while *BRAF* mutations and *RAF1* mutations seemed to have poor prognosis because of medium or severe mean and peak gradients across the obstruction of pulmonary valve or the left ventricular outflow tract.

**TABLE 3:** Catheter or surgery-based interventions and follow-up clinical data.

Case	Age (m)	Genotype	Procedure type	Follow-up (y)	PV* (mmHg)	PV# (mmHg)	LVOT* (mmHg)	LVOT# (mmHg)	RVOT* (mmHg)	RVOT# (mmHg)
Case 3	10	*SOS1*	PBPV, right ventricular outflow tract dredge + pulmonary valve formation (GOTX bicuspid valve) + atrial septal defect repair	2	99	45	—	—	—	—
Case 4	15	*RIT1*	PBPV, right ventricular outflow tract dredge + pulmonary valve formation (GOTX bicuspid valve) + atrial septal defect repair	1	112	22	—	—	43	19
Case 5	16	*SOS1*	PBPV	1.5	64	42	—	—	—	—
Case 8	36	*RAF1*	Morrow, mitral valvuloplasty, left ventricular + right ventricular outflow tract dredge	1.25	—	—	75	80	33	11
Case 9	96	*PTPN11*	Percutaneous closure of atrial septal defect	0.5	—	—	—	—	—	—
Case 10	20	*PTPN11*	Percutaneous closure of atrial septal defect	1	23	9	—	—	—	—
Case 13	23	*BRAF*	right ventricular outflow tract dredge + pulmonary valve formation (GOTX bicuspid valve) + atrial septal defect repair	1.5	110	62	—	—	83	29
Case 14	49	*SOS2*	PBPV	0.25	95	20	—	—	—	—
Case 17	108	*BRAF*	Left ventricular outflow tract dredge + mitral valve replacement	No	—	—	128	—	—	—
Case 21	36	*PTPN11*	PBPV, pulmonary valve formation + ASD + VSD repair + PDA ligation	2	71	57	—	—	76	64

Abbreviations*, preoperative peak pressure; #, the last postoperative follow-up peak pressure; PV, pulmonary valve; —, no pressure or loss to follow-up; RVOT, right ventricular outflow tract; LVOT, left ventricular outflow tract.

**FIGURE 5:**
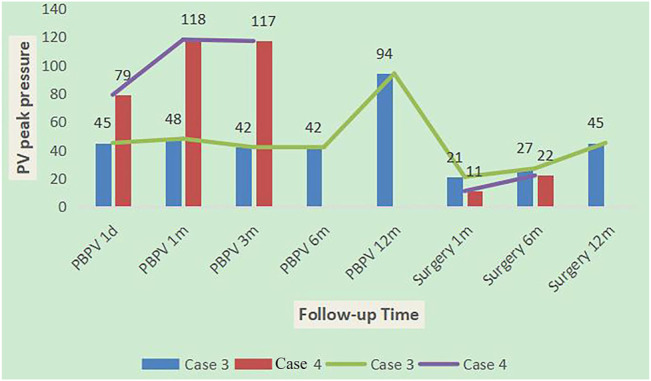
Follow-up results in case 3 and case 4.

Among ten NS patients, cases 3, 4, and 21 had common features of pulmonary valve dysplasia with stenosis, and each experienced interventions twice. The first interventions for cases 3 and 4 were PBPV, but afterward, the pulmonary valve peak differential pressures increased gradually at 1 year of follow-up, and both patients then experienced a second intervention [right ventricular outflow tract dredge + pulmonary valve (GOTX bicuspid valve) formation]. After 1 year of follow-up, the peak differential pressure of the pulmonary valve increased gradually, but more slowly than PBPV (Figure 5). The first intervention of case 21 was pulmonary valve formation, ASD and VSD repair and PDA ligation in another hospital, but 2 years later, the pulmonary valve peak differential pressure was 71 mmHg, so PBPV was performed in our hospital. After 2 years of follow-up, the peak differential pressure of the pulmonary valve was 57 mmHg. From the clinical data, we speculate that PBPV was not a good choice for NS patients. Three patients had only complex surgery. Case 13 and 17 with *BRAF* mutation showed pulmonary valve stenosis or HCM. Case 13 underwent right ventricular outflow tract dredge, pulmonary valve formation (GOTX bicuspid valve) and ASD repair. The pulmonary valve peak differential pressure decreased from 110 to 62 mmHg after 1 and a half years. Case 17 was lost to follow-up. Before the operation, the patient had severe left ventricular outflow tract stenosis and mitral regurgitation. Case 8, with a *RAF1* mutation, showed severe obstructive HCM and mitral regurgitation, then underwent Morrow and mitral valvuloplasty procedures. However, the left ventricular outflow tract peak differential pressure still increased to 80 mmHg after 15 months, which was not a good outcome. Thus, for HCM in NS patients, surgery appears to lead to poor outcomes.

## Discussion

NS is the second most common syndromic cause of congenital heart disease, exceeded in prevalence only by trisomy 21 (Marino et al., 1999). Cardiac defects problems are the most common reasons that prompted these patients to see a doctor. There are several cardiovascular phenotypes abnormalities in NS. The most common phenotypes are pulmonary valve dysplasia with stenosis (50–62%), HCM (20%), ASDs (6–10%) and unusual electrocardiographic patterns (50%). Other cardiac abnormalities were also noted, like peripheral pulmonary stenosis, ventricular septal defect, mitral valve abnormalities, atrioventricular canal, aortic valve stenosis, aortic coarctation and coronary artery anomalies (Sanchez-Cascos, 1983; Patton, 1994; Shaw et al., 2007). Rarely, tetralogy of Fallot or patent ductus arteriosus are observed in NS. Often, patients display complex cardiac phenotypes with multiple defects such as pulmonary valve stenosis and aortic valve stenosis or CHD and HCM (Prendiville et al., 2014). This result is not entirely consistent with our clinical data from a single center. In this study, we found that the most commonly detected abnormality was pulmonary valve dysplasia with stenosis (68.2%), followed by ASD (50%), biventricular hypertrophy (31.8%), left HCM (27.3%), mitral regurgitation (27.3%), PFO (27.3%), both coronary artery anomalies and pulmonary branches stenosis (9.1%) and left ventricle globose dilation (4.5%). In particular, the incidence of ASD was significantly higher than that reported in literature, due to the small cohort. Also, we observed left ventricle globose dilation, which has not been reported in previous literature. The presentation was similar to dilated cardiomyopathy.

With the development of whole-exome sequencing, genetic testing is very useful for diagnosis of NS or NS-like disorders. If a patient has a mild or atypical presentation, genotyping could establish the diagnosis. To establish better treatment and management, it is necessary to look for genotype-phenotype associations with respect to NS-associated CHD phenotypes. There are eight known genotypes that cause NS, in the genes *PTPN11*, *SOS1*, *RAF1*, *KRAS*, *NRAS*, *BRAF*, *SHOC2*, and *CBL* (Romano et al., 2010). Recently, however, studies have found several novel genes associated with NS, like *RIT1*, *RASA2*, *A2ML1*, *SOS2* and *LZTR1* (Aoki et al., 2016). In our study, we detected variants in seven genes associated with NS. Mutations in four known genes (*PTPN11*, *RAF1*, *SOS1*, *BRAF*) and three novel genes (*RIT1*, *SOS2*, *LZTR1*) and, overall, *PTPN11* (27%) and *RAF1* (27%) were the most common.

*PTPN11* encodes the Src homology-2 domain containing protein tyrosine phosphatase (Tartaglia et al., 2001; Tartaglia et al., 2002; Tartaglia et al., 2010). Studies suggest that 50% of cases of NS are caused by missense, gain-of-function mutations in *PTPN11* (Tartaglia et al., 2001; Maheshwari et al., 2002; Tartaglia et al., 2002). NS in patients with *PTPN11* mutations is more likely be associated with pulmonary valve stenosis or ASD (ostium secundum type) (Yoshida et al., 2004; Sznajer et al., 2007). This is consistent with the findings of our study. Six patients had pulmonary valve stenosis or ASD with *PTPN11* mutations. *RAF1* was another commonly mutated gene, often associated with HCM and negatively associated with pulmonary valve stenosis (Pandit et al., 2007). NS cases with *RAF1* mutations were estimated to comprise 5–15% of the population (Pandit et al., 2007; Razzaque et al., 2007). *RAF1* mutations prevent the phosphorylation of Ser259, causing a gain of function of the kinase, contributing to the autoinhibition of RAF1, affecting the activation loop and reducing the activity of the kinase (Pandit et al., 2007; Tidyman and Rauen, 2009). Our study found that six patients had *RAF1* mutations, and the cardiac abnormalities included not only HCM (obstructive or non-obstructive), but also ASD and mitral regurgitation. *SOS1* mutations most often result in pulmonary valve stenosis, much like *PTPN11* mutations (Lepri et al., 2011). Missense mutations in *SOS1* account for roughly 10% of cases of NS (Tidyman and Rauen, 2009; Tartaglia et al., 2010), close to our study results (9%). The protein product of *SOS1* is a guanine nucleotide exchange factor that activates RAS proteins by displacing GDP, enabling GTP to bind through mass action (Egan and Weinberg, 1993; Takai et al., 2001). Other than pulmonary valve stenosis, *SOS1* was associated with pulmonary valve dysplasia, biventricular hypertrophy and ASD in our study. *BRAF* mutations were rare, but have also been reported in individuals meeting clinical diagnostic criteria for NS. *BRAF* mutation-associated NS is characterized by HCM (Razzaque et al., 2007; Nyström et al., 2008; Sarkozy et al., 2009). However, *BRAF* predisposed patients to both HCM and valve abnormalities and accounted for roughly 14% of cases of NS in our study.

Three novel genotypes were also related to cardiac abnormalities. The mutations in *RIT1* in NS patients are located in its G1 domain (p.S35T) and in the switch I region that is included in its G2 domain (p.A57G) (Aoki et al., 2016). Chen et al. (Chen et al., 2014) identified *RIT1* mutations in five out of 27 (18.5%) individuals with NS. Bertola et al. (Bertola et al., 2014) and Gos et al. (Gos et al., 2014) identified *RIT1* mutations in six out of 70 (8.6%) individuals and four out of 106 individuals (3.8%), respectively. Aoki et al. (Aoki et al., 2016) suggested that the frequency of *RIT1* mutations can be estimated at ∼5% in patients with NS. Our study found that 3/22 (14%) NS patients had *RIT1* mutations, significantly higher than the data reported in the literature. This may be related to the ethnicity of the population and the small number of patients, which requires further study. *RIT1* mutations predispose patients to both HCM and valve abnormalities (Gelb et al., 2015), while our findings related to cardiac malformation were pulmonary valve dysplasia with stenosis, ASD and PFO. To date, few studies have reported cardiac abnormalities related to *SOS2* and *LZTR1. SOS2* is homologous to *SOS1*, and the identified variants, p.M267K and p.T376S, were located in the DH domain of *SOS2* (Aoki et al., 2016)*.* In our study, we found that pulmonary valve stenosis with regurgitation was related to cardiac malformation, which was similar to *SOS1*. Another novel genotype in *LZTR1* had a unique cardiac phenotype, left ventricle globose dilation, with a presentation similar to dilated cardiomyopathy. *LZTR1* is located within the 3-Mb-long region that is often deleted in 22q11 deletion syndrome patients (Kurahashi et al., 1995). Some studies have found that somatic and germline mutations in *LZTR1* were associated with glioblastoma multiforme (Frattini et al., 2013) and multiple schwannomas (Piotrowski et al., 2014). Our study first reported this unique cardiac malformation related to *LZTR1*. Additional studies should be performed on mutational and cardiac malformation analyses.

A total of ten cases underwent catheter or surgery-based interventions in our study. Major interventions were PBPV, percutaneous closure of ASD and complex surgery. Cardiac malformations that require management were severe pulmonary valve stenosis, obstructive HCM and congenital heart disease of hemodynamic significance. In this group NS patients, we tried to describe the trend of prognosis for the correlations between genotypes and cardiac phenotypes according to the follow-up results of the last echocardiogram after catheter or surgery-based interventions. *SOS1*, *RIT1*, *PTPN11*, and *SOS2* mutations showed common cardiac abnormalities such as pulmonary valve stenosis. For mild pulmonary valve stenosis in NS, stenosis tends to be nonprogressive and unlikely to require intervention. For moderate or severe obstruction, like *BRAF* mutations and *RAF1* mutations, the rates of intervention are higher (∼50 and 100%, respectively) (Linglart and Gelb, 2020). McCrindle reported that the standard intervention using PBPV was often not as successful as was typical for pulmonary valve stenosis because of frequent dysplasia with commissural fusion and thickened pulmonary valve leaflets (McCrindle, 1994). We found that some of the cases had good results via PBPV, such as case 5 and case 14 with mutations in *SOS1* and *SOS2*, respectively, but this may be related to our short follow-up period. Cases like case 3 and 4, with mutations in *SOS1* and *RIT1*, respectively, did not show good results after the first PBPV, so they underwent surgical valvotomy again, which appeared to be highly successful. However, case 21 with a mutation in *PTPN11* first underwent surgical valvotomy with poor results at 2 years of follow-up, then underwent PBPV again, which appeared to be successful. Holzmann et al. (Holzmann et al., 2018) reported that the pulmonary gradient remained unfavorably elevated in 80% of patients with NS after the initial PBPV and reintervention was necessary in up to 65% of cases (Prendiville et al., 2014). The clinical data from our center suggests that multiple interventions may be required for NS patients with severe pulmonary stenosis, whether PBPV or valvulotomy. Additionally, when the first intervention fails, the second intervention appears to be highly successful. Additional follow-up data are needed.

.Obstructive HCM in NS is another complex problem in the clinic. It can lead to severe heart failure and arrhythmias with progression of HCM. Most patients prefer to choose surgery. In our study, two genotypes in *RAF1* and *BRAF* resulted in a common cardiac abnormality, obstructive HCM. These two genotypes seemed to have poor prognosis. While myectomy for HCM in a patient with NS is initially useful, with time, the left ventricular outflow tract may become obstructed again. Wilkinson et al. (Wilkinson et al., 2012) reported that patients with NS with HCM have a worse risk profile at presentation compared with other children with HCM, resulting in significant early mortality (22% at 1 year). Thus, heart transplantation may be a preferrable approach. It is encouraging that much progress has been made in our understanding of the molecular genetic causes of NS, and novel therapeutic drugs have been given to patients. To date, there are some publications describing the use of RAS pathway inhibition as useful for NS (Andelfinger et al., 2019; Mek Inhibitor Reverses Hypertrophic Cardiomyopathy, 2019; Dori et al., 2020; Meisner et al., 2021; Mussa et al., 2021; Kontaridis et al., 2022). Trametinib, a MEK-inhibitor approved for treatment of RAS/MAPK-mutated cancers, is an emerging treatment option for HCM in NS. Trametinib was given to two infants with the severe, rapidly progressive form of HCM with *RIT1* mutations and no adverse effects have been observed as of yet (Andelfinger et al., 2019). However, further study of the effects of trametinib and other pathway inhibitors for pulmonary valve stenosis or HCM in NS is necessary.

## Study Limitations

The retrospective design of the study had some limitations. We tried to grade the correlations between genotypes and cardiac phenotypes according to the follow-up results of the last echocardiogram after catheter or surgery-based interventions. However, the relatively small size of the cohort may have biased the results. Since each genotype group consisted of only a few individuals, we limited our analysis to describing prognostic trends for cardiac phenotypes within each group.

## Conclusion

Molecular diagnoses are enabling earlier and more precise identification of patients with a subtle or atypical presentation of NS. The identification of causal genes in NS patients has enabled the evaluation of genotype-cardiac phenotype relationships and may be beneficial to the development of therapeutic approaches. Although there are many extracardiac malformations, in some cases, progressive cardiac malformations still need to be managed, which will lead to better prognoses for patients with this disorder. In addition, NS-specific therapeutic medications require further research.

## Data Availability

The original contributions presented in the study are included in the article/supplementary material, further inquiries can be directed to the corresponding authors.
